# Using spatial uncertainty to manipulate the size of the attention focus

**DOI:** 10.1038/srep32364

**Published:** 2016-09-01

**Authors:** Dan Huang, Linyan Xue, Xin Wang, Yao Chen

**Affiliations:** 1 School of Biomedical Engineering, Shanghai Jiao Tong University, Shanghai, 200240, China; 2 School of Quality and Technical Supervision, Hebei University, Baoding, 071002, China

## Abstract

Preferentially processing behaviorally relevant information is vital for primate survival. In visuospatial attention studies, manipulating the spatial extent of attention focus is an important question. Although many studies have claimed to successfully adjust attention field size by either varying the uncertainty about the target location (spatial uncertainty) or adjusting the size of the cue orienting the attention focus, no systematic studies have assessed and compared the effectiveness of these methods. We used a multiple cue paradigm with 2.5° and 7.5° rings centered around a target position to measure the cue size effect, while the spatial uncertainty levels were manipulated by changing the number of cueing positions. We found that spatial uncertainty had a significant impact on reaction time during target detection, while the cue size effect was less robust. We also carefully varied the spatial scope of potential target locations within a small or large region and found that this amount of variation in spatial uncertainty can also significantly influence target detection speed. Our results indicate that adjusting spatial uncertainty is more effective than varying cue size when manipulating attention field size.

Spatial-based attention facilitates perception of stimuli within specific regions in the visual field and typically increases the responses of those neurons whose receptive fields overlap the attended location. The spatial extent of this attention-mediated facilitation or enhancement, known as the attention field, is an important characteristic of visuospatial attention. Many theoretical models of visuospatial attention, such as spotlight^1^,^2^, gradient^3^, zoom lens^4^,^5^, and normalization^6^,^7^, have hypothesized different features about the attention field. Some studies have investigated the spatial distribution of the attention field and proposed that it has a Mexican hat distribution^8^,^9^,^10^,^11^. A recent neuroimaging study even measured the topography of the attention field and revealed that it scaled with eccentricity and varied across visual areas, with a structure which can be well modeled by an enhancing Gaussian center with a suppressive surround^12^. In addition to its structure, another feature of the attention field is also critical: its flexibility in size. A straightforward inference from the zoom lens model is as follows: the smaller the attention field, the higher the processing efficiency within that region. Psychophysical studies have revealed that automatic attention captured by distractors was reduced when the attention field was narrowed^13^,^14^,^15^. Additionally, a neuroimaging study^16^ showed that while the activated visual cortex regions increased with the size of the attention field, the level of neural activity in a given cortical subregion decreased. The behavioral performance of the subjects also had a negative correlation with the spatial extent of the attended area. Reynolds and Heeger^6^ argued that the variety and complexity of the reported visuospatial attention modulations may be ascribed to the uncontrolled attention field size in experimental protocols. Their normalization model made a prediction about the cortical responses to stimulus contrast. They proposed that attention increases neuronal responses by a multiplicative response gain when the stimulus is large and the attention field is small. Conversely, when the stimulus is small and the attention field is large, attention results in a leftward shift of the contrast-response (contrast gain). This prediction was soon verified by psychophysical and electrophysiological studies^17^,^18^.

The significance of the attention field has been recognized in many spatial attention studies. Finding effective ways to finely manipulate attention field size is very important for the design of a practical experiment. Two methods for manipulating the spatial extent of attention have been used in previous studies: changing the size of the cue orienting the attention focus and varying the spatial uncertainty of the target position.

In experiments using cues to orient the focus of spatial attention, if a small cue induces a constricted attentional scope, it should lead to a stronger attentional effect than a large cue, which provokes less focused attention. Based on this idea, several psychophysical studies^19^,^20^,^21^,^22^ used spatial cues of different sizes to indicate the possible position of an impending stimulus and then measured the subjects’ reaction times (RTs) to detect the target. They found a positive relationship between cue size and the subsequent RTs of target detection, which they named the cue size effect^22^, and claimed that it was empirical support for the zoom lens model. This reported cue size effect is very appealing and implies that the spatial extent of attention can be adjusted by merely varying the cue size. However, later research^23^ found that the influence of cue size depended heavily on the experimental design and on the cue-target stimulus onset asynchrony (SOAs). Thus, the cue size effect was affected by many factors.

Spatial uncertainty, having incomplete or unknown information about the target location, has a great impact on visual search and detection. When Eriksen and James^4^ proposed the zoom lens model of attention, they also put forth the notion that when spatial uncertainty increases, the size of the attention focus increases, with a concomitant reduction in processing efficiency within the attended region. Thus, changing the spatial uncertainty may experimentally vary the attention field size. The neuroimaging study mentioned above, which provided physiological evidence for the zoom lens model, manipulated the attention field size by varying the number of cues indicating the possible locations of the target^16^. Recently, another neuroimaging experiment found that the attention field size, quantified as the spread of the spatial distribution of cortical response differences, was larger when stimuli were presented with spatial uncertainty^18^. Furthermore, several electrophysiological experiments showed that the activity of saccade-related superior colliculus neurons in monkeys decreased as the spatial uncertainty of the target increased while they were performing a saccade task^24^,^25^, which may be suggestive of the neuronal mechanism of spatial uncertainty modulation.

Although studies on spatial attention should manipulate the attention field size, methods of achieving this goal have not been thoroughly investigated. Our study systematically examined and compared two previously used methods of attention field size manipulation: varying the cue size and changing the spatial uncertainty. Comparisons between different levels of spatial uncertainty and different cue sizes were conducted in our experiments. We found that smaller cue size does not necessarily lead to higher processing efficiency within the cued area, and that spatial uncertainty reduction always causes a decrease in target detection reaction time. We also demonstrated that the spatial extent of attention can be finely manipulated by adjusting the spatial scope of potential target locations within a small region.

## Experiment 1a

### Subjects

Eight young adults (20–34 years old, 4 male, 4 female) who were students at Shanghai Jiao Tong University provided informed consent to participate in this study. All subjects had normal or corrected-to-normal vision. All experimental procedures were approved by the Ethical Committee of Shanghai Jiao Tong University and conformed to the guidelines of the Declaration of Helsinki.

### Apparatus

Visual stimuli were presented on a 24-inch liquid crystal display (LCD) monitor (BenQ xl2411t, Taipei, Taiwan, 1920 × 1080 pixels, 100 Hz refresh rate) positioned 57 cm from the subject. The mean luminance of the screen was 15.5 cd/m^2^. The head position of the subject was held in place using a chin rest, and eye position was monitored using an infrared imaging-based eye tracker (Tobii X60; Tobii Technology AB, Stockholm, Sweden). MATLAB (MathWorks) with Psychtoolbox was used to control stimulus presentation and collect manual reaction time (RT) data. Data were analyzed using the Statistical Package for Social Sciences (SPSS, Inc.) and OriginPro software (OriginLab Corporation).

### Stimuli and procedure

**Figure 1:**
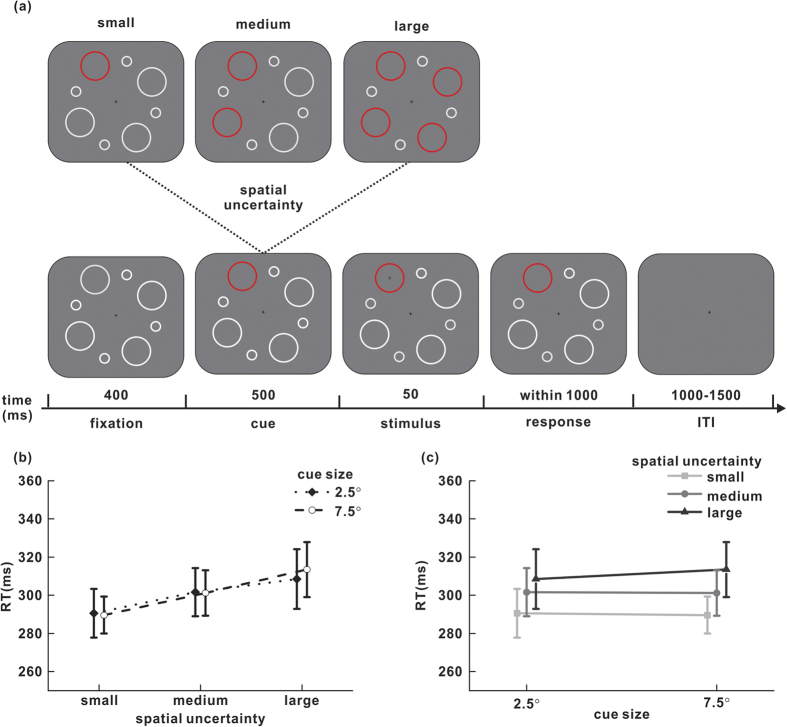
(**a**) The sequence of events in a typical trial. Each trial began with the appearance of eight evenly distributed white rings around the fixation cross, followed by a cue period in which one, two or four rings changed color to red to instruct the subjects regarding the potential locations of the target. Different numbers of red rings represented different levels of the target’s spatial uncertainty: one for small, two for medium, and four for large. The diameters of the cued rings were either 2.5° or 7.5° in each trial. In 80% of the trials, a single dark dot target appeared at the center of one of the cued rings for 50 ms. Observers were required to press a key as soon as possible when they detected the target. ITI, inter-trial interval. (**b,c**) Mean RTs of all eight participants in experiment 1a, plotted for each cue size and each spatial uncertainty level. Short vertical bars represent the standard error of the mean across participants.

Subjects were instructed to fixate on a black cross (0.3° × 0.3°; Fig. 1a) at the center of the screen, which was presented throughout the entire experiment. Eight white thin rings, evenly spaced around the central cross with 10° of eccentricity, were displayed at the beginning of each trial. The rings alternated in diameter, 7.5° or 2.5°. After 400 ms of fixation, one, two or four of the rings of the same size changed color to red as a visual cue. To minimize the effect of changing the ring color on target detection, the rings in red and white had the same luminance (17.5 cd/m^2^). After an interval of 500 ms, there was an 80 percent chance that a dark stimulus (a dot, 0.4° in diameter, 11.5 cd/m^2^) would appear at the center of one of the cued rings for 50 ms. The participants were instructed to press key ‘5’ as soon as possible when they detected the appearance of the target and to not respond during the trials when no target stimulus appeared (catch trials). Reaction time (RT), the time between target onset and response emission, was recorded for further analysis. The trial ended once the subject made a response, or the rings disappeared. Another trial began after an inter-trial interval (ITI) randomized from 1000 to 1500 ms. The diameter of each ring differed from that of the previous trial, e.g., the diameter of a ring was 7.5° in the next trial if its diameter in the previous trial was 2.5°, and vice versa.

Each subject completed eight to ten blocks. Within each block, the subject was instructed to correctly complete 60 trials, including three levels of spatial uncertainty and two cue sizes, in a random order. The subjects could hold their fixation within a 3° diameter window while performing the task. Those trials that had fixation breaks, false alarms or reaction times longer than 1 s or shorter than 150 ms were considered incorrect and were repeated at the end of each block.

### Results and discussion

Data were analyzed by two-way repeated-measures analysis of variance. The two factors were spatial uncertainty (small, medium or large) and the size of the ring cue (2.5° or 7.5°). Trials with an error were rare (less than 2%), and the false alarm rates in catch trials among the different conditions were analyzed (small spatial uncertainty: 2.5° cue size, 2.03%; 7.5° cue size, 1.26%; medium spatial uncertainty: 2.5° cue size, 0.57%; 7.5° cue size, 0.74%; large spatial uncertainty: 2.5° cue size, 1.21%; 7.5° cue size, 0.78%). No significant influences of cue size, spatial uncertainty or their interaction on false alarm rates were found (F_2,14_ = 0.48, P = 0.631 for spatial uncertainty; F_1,7_ = 1.91, P = 0.209 for cue size; F_2,14_ = 0.30, P = 0.747 for their interaction).

There was a significant influence of spatial uncertainty on RT (F_2,14_ = 19.65; P < 0.001). As shown in Fig. 1b, RT was positively correlated with spatial uncertainty levels. The inverse relationship between spatial uncertainty and detection speed was very similar for trials with either a small cue (291 ms, 302 ms and 308 ms for small, medium and large spatial uncertainty, respectively) or a large cue (290 ms, 301 ms and 313 ms for small, medium and large spatial uncertainty, respectively). Cue size had no significant impact on RT (F_1,7_ = 0.33; P = 0.586), regardless of the spatial uncertainty level (Fig. 1c). The interaction between spatial uncertainty and cue size was also not significant (F_2,14_ = 1.04; P = 0.379). These data suggest that RT varies with spatial uncertainty instead of cue size.

## Experiment 1b

In spatial attention research, two types of indicators orienting the attention focus are commonly used: peripheral indicators, such as the ring cue changing color in experiment 1a, and symbolic central pointers. In an event-related study, these two cueing methods differentially affected sensory processing at an indicated location^26^. Experiment 1b was conducted to examine the effect of spatial uncertainty and ring cue size when a central pointer indicated the cued positions.

### Subjects and procedure

Eight subjects (age 22–34 years, 3 male, 5 female) who were students at Shanghai Jiao Tong University provided informed consent to participate in this study. All subjects had normal or corrected-to-normal vision. All experimental procedures were approved by the Ethical Committee of Shanghai Jiao Tong University and conformed to the guidelines of the Declaration of Helsinki.

**Figure 2:**
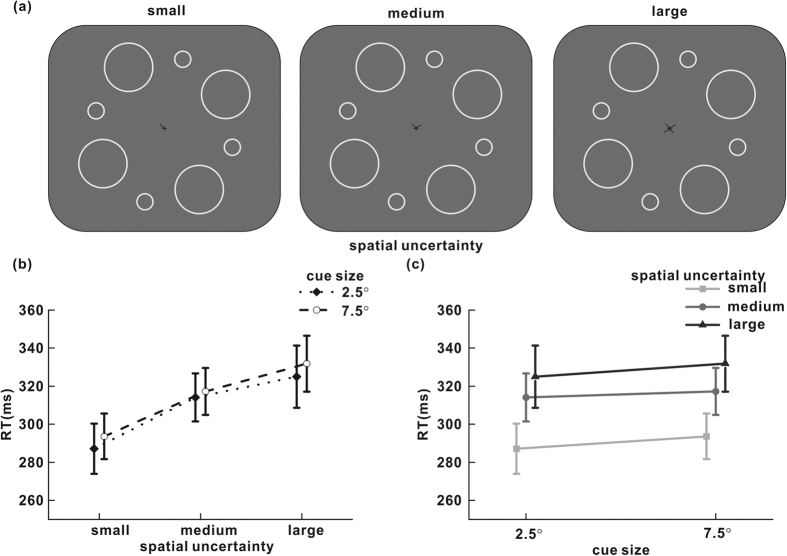
(**a**) Central cueing was used in experiment 1b. In the cue period, one, two or four black lines appeared near the fixation cross, pointing to the surrounding rings where the target would be presented. Different numbers of lines represented different levels of the target’s spatial uncertainty: one for small, two for medium, and four for large. (**b,c)** Mean RTs of all eight participants in experiment 1b, plotted for each cue size and each spatial uncertainty level. Short vertical bars represent the standard error of the mean across participants.

All settings were identical to those used in experiment 1a, except that no color change occurred. Symbolic central lines were used (Fig. 2a) to indicate the rings that required attention.

### Results and discussion

Data were analyzed by two-way repeated-measures analysis of variance. The two factors were spatial uncertainty level (small, medium or large) and the size of the ring cue (2.5° or 7.5°). Trials with an error were rare (less than 2%), and the false alarm rates among the different conditions were analyzed (small spatial uncertainty: 2.5° cue size, 2.72%; 7.5° cue size, 2.75%; medium spatial uncertainty: 2.5° cue size, 0.38%; 7.5° cue size, 1.25%; large spatial uncertainty: 2.5° cue size, 1.02%; 7.5° cue size, 0.66%). No significant influences of cue size, spatial uncertainty or their interaction on false alarm rates were observed (F_2,14_ = 1.25, P = 0.316 for spatial uncertainty; F_1,7_ = 0.67, P = 0.441 for cue size; F_2,14_ = 0.29, P = 0.752 for their interaction).

For RT, the influence of spatial uncertainty was significant (F_2,14_ = 42.87; P < 0.001). As indicated in Fig. 2b, RT increased as a function of the spatial uncertainty level. This relationship was present regardless of whether the cue size was 2.5° (287 ms, 314 ms and 325 ms for small, medium and large spatial uncertainty, respectively) or 7.5° (294 ms, 317 ms and 332 ms for small, medium and large spatial uncertainty, respectively). Cue size had a weak but significant impact on RT (F_1,7_ = 7.64; P = 0.028), which was not observed in experiment 1a. The results of experiment 2b confirm that the spatial uncertainty of the target influences its detection speed and also show the influence of cue size on target detection. However, the impact of cue size on RT is much weaker than that of spatial uncertainty.

## Experiment 2a

Experiment 1 demonstrated how spatial uncertainty could significantly impact target detection reaction time and also demonstrated that cue size was likely to be a less effective way to manipulate attention field size. However, it was not clear whether the spatial uncertainty effect could still be observed if the size of the region where a target stimulus would appear was adjusted. Experiment 2a was conducted to answer that question.

### Subjects and procedure

Eight subjects (age 19–33 years, 4 male, 4 female) who were students at Shanghai Jiao Tong University provided informed consent to participate in this study. All subjects had normal or corrected-to-normal vision. All experimental procedures were approved by the Ethical Committee of Shanghai Jiao Tong University and conformed to the guidelines of the Declaration of Helsinki.

**Figure 3:**
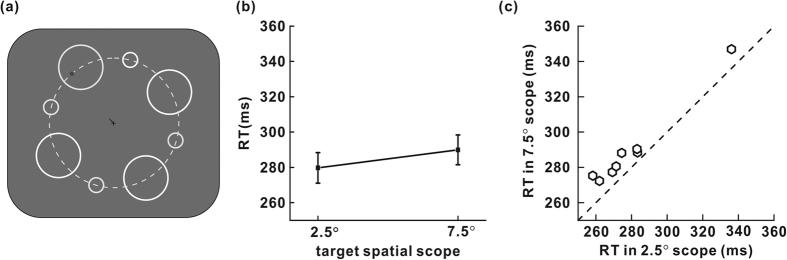
(**a**) Typical stimulus arrangement in experiment 2a. The target was presented randomly on a virtual, iso-eccentric arc (the dashed, white ring, not displayed during the experiments) within the cued ring. The scope of the potential target location was either 2.5° or 7.5° in each trial. (**b**) Mean RTs of all subjects are shown for the two spatial scope sizes (2.5° and 7.5°) of potential target locations. (**c**) The effect of the spatial scope of potential target locations on RTs for individual observers. Each hexagon represents RTs in 2.5° scope vs. 7.5° scope.

All settings were identical to those used in experiment 1b, except that (1) for each trial, one line near the central cross point of a ring restricted the potential positions of the upcoming target, and (2) the target was presented at positions randomly on a virtual, iso-eccentric arc of radius (10°) within the ring instead of at its center (Fig. 3a). Narrowing the spatial scope of the potential target locations deduced the spatial uncertainty level. Each subject completed ten blocks. Within each block, the subject was instructed to correctly complete 40 trials, including different spatial scopes of the potential target locations in a random order.

### Results and discussion

Data were analyzed by one-way repeated-measures analysis of variance (two spatial scopes of the potential target locations). The false alarm rates in these two conditions (1.41% for small spatial scope, 2.00% for large spatial scope) were not significantly different (F_1,7_ = 0.55, P = 0.483).

Figure 3b shows the mean RTs of all eight participants in experiment 2a. For RTs, the effect of the spatial scope was significant (F_1,7_ = 57.79; P < 0.001). As seen in Fig. 3b, for the trials where the target appeared within a spatial scope of 2.5°, the RTs were shorter than the RTs of trials with a large spatial scope. The difference between the mean RTs of the two groups was 10 ms (280 ms for small spatial scope, 290 ms for large spatial scope). Variation in spatial uncertainty led to changes in RT. Although the RT discrepancy of the two groups was not large, the impact of spatial scope was very robust. As shown in Fig. 3c, all eight subjects responded faster when the spatial scope of the potential target locations was small. One reason for not seeing large discrepancy in RTs may be that changing the spatial scope of the potential target locations from 2.5° to 7.5° in the experiment can only result in a small variation in the attention field size. Consequently the corresponding RTs showed little modulation.

## Experiment 2b

In experiment 2a, the regions where a target stimulus would appear were indicated by rings of different sizes throughout the trial. It was possible that the size of the ring and spatial uncertainty conjointly affected the detection of the target. To observe the solely influence of spatial uncertainty on target detection, we investigated the modulation of varying spatial uncertainty on target detection without the appearance of surrounding rings in experiment 2b.

### Subjects and procedure

Eight subjects (age 19–33 years, 7 male, 1 female) who were students at Shanghai Jiao Tong University provided informed consent to participate in this study. All subjects had normal or corrected-to-normal vision. All experimental procedures were approved by the Ethical Committee of Shanghai Jiao Tong University and conformed to the guidelines of the Declaration of Helsinki.

**Figure 4:**
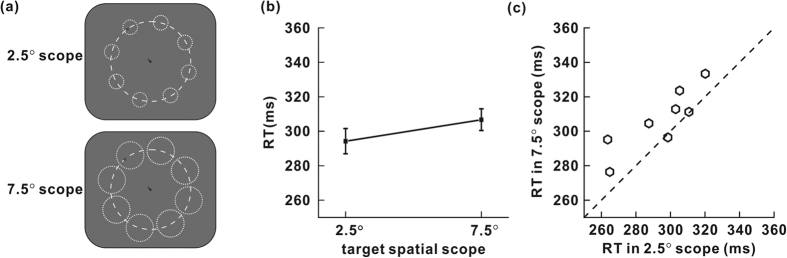
(**a**) Typical stimulus arrangement in experiment 2b. The target was presented randomly on a virtual, iso-eccentric arc (the dashed, white ring, not displayed during the experiments) within one of the regions (the dotted, white rings, not displayed during the experiments) cued by a black line appeared near the fixation cross. The scope of the potential target location was either 2.5° or 7.5° within each block. (**b**) Mean RTs of all subjects are shown for the two spatial scopes (2.5° and 7.5°) of potential target locations. (**c**) The effect of the spatial scope of potential target locations on RTs for individual observers. Each hexagon represents RTs in 2.5° scope vs. 7.5° scope.

All settings were identical to those used in experiment 2a, except that (1) no ring was presented on the screen (Fig. 4a), and (2) we used a between-block design instead of a within-block one. Each subject randomly completed ten blocks in two conditions, which the spatial extent of the potential target locations was kept at either 2.5° or 7.5° (Fig. 4a). Within each block, the subject was instructed to correctly complete 60 trials.

### Results and discussion

Data were analyzed by one-way repeated-measures analysis of variance (two spatial scopes of the potential target locations). The false alarm rates in these two conditions (2.04% for small spatial scope, 0.20% for large spatial scope) were not significantly different (F_1,7_ = 1.73, P = 0.229).

Figure 4b shows the mean RTs of all eight participants in experiment 2b. For RTs, the effect of the spatial scope was significant (F_1,7_ = 11.13; P = 0.012). As seen in Fig. 4b, for the trials where the target appeared within a spatial scope of 2.5°, the RTs were shorter than the RTs of trials with 7.5° spatial scope. The difference between the mean RTs of the two groups was 13 ms (294 ms for small spatial scope, 307 ms for large spatial scope). As shown in Fig. 4c, six out of eight subjects responded faster when the spatial scope of the potential target locations was small.

The results of experiment 2b demonstrate that merely varying the scope of potential target locations can result in significant modulation on target detection speed.

## General discussion

In this study, we provide evidence for three main findings: (1) detection speed is strongly influenced by spatial uncertainty, (2) cue size have a less robust impact on detection speed, and (3) varying the spatial scope of the potential target locations can significantly impact target detection. The detection difficulty levels in our experiments were similar across different conditions as the performance accuracies in all of these conditions were extremely high (above 98%). Therefore, detection speed reflected processing efficiency in our experiments as follows: the longer the RT, the lower the processing efficiency. Given that RT has been widely used to investigate the spatial extent of the attention field in previous studies, our results suggest that the size of the attention field is more effectively manipulated by spatial uncertainty than cue size.

The failure to find a consistent cue size effect in experiment 1 is intriguing. Previous research on the cue size effect^20^,^21^,^22^,^27^ mainly focused on psychophysical works with tasks, which required participants to detect whether a single element appeared at the center of a cue. To the best of our knowledge, there was no further electrophysiological or neuroimaging evidence. Furthermore, another psychophysical study^23^ also investigated the cue size effect with different SOAs and found that the cue size effect was not universally present. However, a ring cue is considered a strong visual stimulus that can affect visual processing. There is evidence that interference from a surrounding ring can occur in a focused attention condition. Studies on paracontrast masking indicate that with different SOAs, polarity, or spatial separation between the target and the ring, a single preceding ring can have a different influence on a subsequent judgment of brightness or contour^28^,^29^. There is also evidence that changing the brightness of a ring cue can alter its impact on future contrast perception^30^, which may be explained by paracontrast masking or a sensory interaction between the ring and the stimulus, instead of by attention modulation^31^. Therefore, the reported cue size effect may be a sensory interaction between the cue and the stimulus instead of attention modulation. The failure to interpret the nature of the cue size effect makes cue size an unsuitable variable to manipulate the spatial extent of the attention field.

There are two types of attention: exogenous and endogenous. The former is passive, reflexive, involuntary, and rises and decays quickly, peaking at approximately 100–120 ms, while the latter is active and voluntary and takes approximately 300 ms to deploy^32^. Spatial uncertainty modulation exists for both exogenous and endogenous attention^18^. According to the results of a previous study, the cue size effect^19^ can last for 60–500 ms after the onset of the cue. Another study also observed the cue size effect at 804 ms SOA^21^. These results suggest that varying the cue size may influence the spatial extent of both exogenous and endogenous attention. Our study used 500 ms SOA, which is suitable for observing and comparing the effects of spatial uncertainty and cue size on voluntary attention field size.

In experiment 2, we presented the target on a virtual, iso-eccentric arc to avoid variations in eccentricity influencing our results. This is because there is evidence that the speed of visual processing increases with eccentricity^33^. Itthipuripat *et al*.^17^ also manipulated the spatial extent of attention by varying the potential location of a target stimulus. However, they did not control for the eccentricity of the target, likely because their target was presented in only 25% of the trials. The observed differences in RTs between the two groups with different scopes may have resulted from different target eccentricity variations in the two groups. Some may argue that performance at iso-eccentric locations also has significant variations: better performance is observed along the horizontal meridian than the vertical meridian and along the lower than the upper vertical meridian. However, we do not believe that this variation was critical for our experiments because the rings in our experiments were evenly distributed across the visual field and were not located at either the horizontal or the vertical meridian.

In all our experiments, spatial uncertainty variation caused by changing the scope or the number of the potential target locations significantly influenced detection speed. Instead of manipulating the spatial extent of their attention focus, the participants may shift a small, fixed size attention beam between these potential target locations. This means there was shorter time stayed at a given location when the spatial uncertainty was larger, which would have led to worse performance in detection. In that case, varying spatial uncertainty solely impacts attention orienting instead of attention focusing. However, previous functional magnetic resonance imaging studies^16^,^34^ which used different numbers of cues to indicate the possible locations of target reported that the extent of activated retinotopic visual cortex increased with the number of the cued areas, with a concomitant decrease in the level of neural activity in a given subregion. A more recent study^18^ showed that participants’ attention field size was larger with than without spatial uncertainty by comparing the spread of cortical activity brought about by attention. Furthermore there was no parametric modulation related to the number of cued locations in frontoparietal areas^34^, which are responsible for controlling attention shifts^35^. These results support that the spatial scope of visual attention zooms with the variation of spatial uncertainty and argue strongly against a shifting strategy. It’s reasonable to claim that varying spatial uncertainty provokes a zooming process that scales the spatial extent and resolution of the attention focus.

In conclusion, we assessed the effectiveness of two approaches to modulate attention field size: changing spatial uncertainty and cue size variation. We demonstrated that spatial uncertainty, not cue size, is the key factor when manipulating attention field size.

## Additional Information

**How to cite this article**: Huang, D. *et al*. Using spatial uncertainty to manipulate the size of the attention focus. *Sci. Rep.* **6**, 32364; doi: 10.1038/srep32364 (2016).
